# Supplementary material for: HIVprotI: an integrated web based platform for prediction and design of HIV proteins inhibitors
Source: J Cheminform. 2018 Mar 9;10:12. doi: 10.1186/s13321-018-0266-y (PMC5845081; doi:10.1186/s13321-018-0266-y)
Supplement: Supplementary file 2 — Additional file 2. Source code of HIVProtI web server. [file 13321_2018_266_MOESM2_ESM.zip › HIVprotI_Source-code/hivprotI/weka-3-6-6/doc/weka/gui/streams/InstanceProducer.html]

InstanceProducer


---


|  |  |  |
| --- | --- | --- |
| **Overview** \| **Package** \| **Class** \| **Tree** \| **Deprecated** \| **Index** \| **Help** \| **Weka's home** | |  |
| **PREV CLASS**   **NEXT CLASS** | **FRAMES**    **NO FRAMES**     **All Classes** |  |
| SUMMARY: NESTED \| FIELD \| CONSTR \| METHOD | DETAIL: FIELD \| CONSTR \| METHOD |  |


---


## weka.gui.streams Interface InstanceProducer

**All Known Implementing Classes:**: InstanceJoiner, InstanceLoader

---

``` public interface InstanceProducer ```

An interface for objects capable of producing streams of instances.

**Version:**
:   $Revision: 1.5 $

**Author:**
:   Len Trigg (trigg@cs.waikato.ac.nz)

---

| **Method Summary** | |
| --- | --- |
| `void` | `addInstanceListener(InstanceListener ipl)` |
| `Instances` | `outputFormat()` |
| `Instance` | `outputPeek()` |
| `void` | `removeInstanceListener(InstanceListener ipl)` |

**Method Detail**

### addInstanceListener

```
void addInstanceListener(InstanceListener ipl)
```

---


### removeInstanceListener

```
void removeInstanceListener(InstanceListener ipl)
```

---


### outputFormat

```
Instances outputFormat()
                       throws java.lang.Exception
```

:   **Throws:**: `java.lang.Exception`

---


### outputPeek

```
Instance outputPeek()
                    throws java.lang.Exception
```

:   **Throws:**: `java.lang.Exception`


---


|  |  |  |
| --- | --- | --- |
| **Overview** \| **Package** \| **Class** \| **Tree** \| **Deprecated** \| **Index** \| **Help** \| **Weka's home** | |  |
| **PREV CLASS**   **NEXT CLASS** | **FRAMES**    **NO FRAMES**     **All Classes** |  |
| SUMMARY: NESTED \| FIELD \| CONSTR \| METHOD | DETAIL: FIELD \| CONSTR \| METHOD |  |


---
